# Serum Vitamin D Levels and Vitamin D Supplement in Adult Patients with Asthma Exacerbation

**DOI:** 10.1155/2016/4070635

**Published:** 2016-11-16

**Authors:** Tadech Boonpiyathad, Teerapol Chantveerawong, Panitan Pradubpongsa, Atik Sangasapaviliya

**Affiliations:** Division of Allergy and Clinical Immunology, Department of Medicine, Phramongkutklao Hospital, Bangkok, Thailand

## Abstract

*Introduction.* Vitamin D deficiency has been linked to an increased risk of asthma exacerbations.* Objective.* This study aimed to compare vitamin D status during the period of severe asthma exacerbations and investigate if vitamin D supplementation improves asthma control.* Methods.* A total of 47 asthmatic patients and 40 healthy subjects participated in this study. Serum 25-hydroxyvitamin D (25(OH)D), asthma control test (ACT) score, and % predicted peak expiratory flow rate were evaluated in the period with and without severe asthma exacerbations. After that, we provided vitamin D2 supplements to the patients with low vitamin D levels for 3 months.* Results.* At the period of asthma exacerbation, the prevalence of vitamin D deficiency and insufficiency was 38.29% and 34.04%. There was no significant difference in the levels of serum 25(OH)D with and without asthma exacerbations but the levels were significantly higher in the healthy group. Serum 25(OH)D levels significantly correlated with ACT score. Moreover, vitamin D2 supplementation improved asthma control in uncontrolled asthma group.* Conclusions.* Hypovitaminosis D was common in asthmatic patients but was not the leading cause of asthma exacerbations. Serum 25(OH)D levels correlated with the ability to control asthma. Improving vitamin D status might be a benefit in uncontrolled asthmatic patients.

## 1. Introduction

Asthma is a chronic inflammatory disease of the lower airway. The prevalence of asthma is approximately 300 million cases in the world and in Thailand it affects approximately 6.9% of adults [1]. Asthma exacerbation is defined as asthma attack. The airway becomes swollen and inflamed and the muscles around the airway contract causing the bronchial tubes to narrow. Symptoms and exacerbations of asthma include breathlessness, coughing, wheezing, and chest tightness [2]. Severe exacerbations of asthma are potentially life-threatening and require prompt care, close observation for deterioration, and frequent treatments [2]. The public health burden of acute exacerbation asthma is an especially important and costly problem. The major cause of exacerbation in children and adults is common respiratory virus [3]. At least one exacerbation is an important risk factor for recurrent asthma exacerbation [4]. Moreover, asthma exacerbation is associated with accelerated loss of lung function [5].

Vitamin D is a fat-soluble vitamin that is important to the body by balancing calcium and bone, innate and adaptive immunity, and homeostasis of many organs [6]. Vitamin D from food or dermal synthesis is inactive and requires enzymatic conversion to become active. Hypovitaminosis D is typically diagnosed by measuring the concentration in blood of 25-hydroxyvitamin D (25(OH)D), the primary circulating form of vitamin D, and then 1,25-dihydroxyvitamin D, the active form of vitamin D, by the enzyme 25-hydroxylase in the liver and kidney [7]. Vitamin D insufficiency is defined as a 25(OH)D level less than 30 ng/mL. Furthermore, vitamin D deficiency is defined as 25(OH)D levels below 20 ng/mL, with a resultant consistent elevation of PTH and reduction in intestinal calcium absorption. People living in tropical countries are exposed to the sun and usually have high levels of vitamin D. However, studies in tropical countries have found the prevalence of vitamin D deficiency to be about 30 to 50% [8, 9]. In Thailand, the prevalence of hypovitaminosis D is nearly more than 50% [10].

Low serum levels of vitamin D have been linked to increased risk of asthma exacerbation in children and adults. Hypovitaminosis D is associated with increased risk of cardiovascular disease, autoimmune disease, cancer, and allergy disease [11, 12]. Vitamin D plays a significant role in the immune system of asthma. It directly affects the adaptive immune system through its effects on Th1, Th2, and regulatory T cells [13]. It promotes peripheral tolerance by inhibiting inflammation and the induction or maintenance of regulatory T cell populations, both IL-10^+^ and FOXP3^+^ [13]. Serum 25(OH)D levels correlated with CD4^+^FOXP3^+^ Treg cells numbers in moderate/severe asthma [14]. This cross-sectional study demonstrated that the frequency of circulating CD4^+^FOXP3^+^ Treg cells is significantly lower in steroid resistance than in steroid sensitive asthmatic patients [14]. Also, it directs the induction of innate antimicrobial mechanisms to efficiently resolve infection, especially respiratory viral infections [15]. Hypovitaminosis D is frequent in asthmatic patients [16, 17]. Notably, vitamin D insufficiency has been associated with severe asthma exacerbations in children and decreased serum vitamin D levels are associated with increased corticosteroid use [18, 19]. Vitamin D deficiency has been associated with the asthma epidemic, obesity in African Americans, westernization of countries with higher-risk populations for asthma, increased airway hyperresponsiveness, lower pulmonary functions, worse asthma control, and possibly steroid resistance [20]. People given vitamin D supplement had fewer asthma attacks needing treatment with oral steroids [21]. The average number of asthma attacks per person per year decreased from 0.44 to 0.28 with vitamin D [21]. Vitamin D reduced the risk of attending hospital with acute exacerbation asthma from 6 per 100 to around 3 per 100 [21].

However, in tropical countries like Thailand, data on the association between serum vitamin D level and asthma exacerbation in adult patients is limited. This study compared serum vitamin D levels during the period of asthma exacerbation and during the periods in stable condition. Also, patients with low vitamin D levels received vitamin D2 supplement for three months and then asthma control and lung function were monitored.

## 2. Methods

This study employed a cross-sectional design. Adults with asthma exacerbation were recruited from the Allergy Clinic and Emergency Department, Phramongkutklao Hospital, Bangkok, Thailand. Bangkok's latitude and longitude are 13°45′ north and 100°31′ east. Severe asthma exacerbation is defined by acute episodes of progressively worsening shortness of breath, coughing, wheezing, and chest tightness. A patient has dyspnea at rest that interferes with conversation [22]. A patient with asthma exacerbation requires treatment with nebulized short-acting beta-2 agonist and oral corticosteroid added to daily asthma medications. ACT is a five-item patient self-assessment tool for asthma control [23]. We enrolled patients (18 years of age or older) with asthma exacerbation and without upper respiratory tract infection. Serum 25(OH)D, asthma control test (ACT) score, and peak expiratory flow rate (PEFR) were measured during the period of severe exacerbation. At least two weeks after the exacerbation, the patients during the period in stable condition (control asthma without inhaled short-acting beta-2 agonist) visited the Allergy Clinic again to check serum vitamin D levels, ACT score, and PEFR. A clinical history of asthma was confirmed by bronchodilator responsiveness (as defined by an improvement in the FEV1 of >200 mL and 12% after administration of four puffs (360 mg) of salbutamol by metered dose inhaler). Participants with significant medical illness other than asthma, those with a history of respiratory tract infection in the past seven days, those with nonadherence to use control medication, and those taking vitamin D supplement were excluded. The control subjects were adults without a history of chronic illness.

During the period of control asthma, if the serum level of 25(OH)D was less than 30 ng/mL, the subjects were treated with the ergocalciferol (vitamin D2) supplement at a dose of 20,000 IU every alternate day. After 3 months, subjects were evaluated again for serum 25(OH)D, ACT score, and PEFR. During 3 months of vitamin D2 supplement, subjects received the same control asthma medication, that is, inhaled long-acting beta-2 agonist plus corticosteroid and leukotriene receptor antagonists. There was no randomization in vitamin D supplement study. The study was approved by the Institutional Review Board, Royal Thai Army Medical Department. All patients provided written informed consent before taking part in the study.

PEFR was measured during forceful exhalation, starting from full lung inflation with the patient standing. Percent predicted PEFR was calculated from standard PEFR value in Thailand [24]. Serum total 25(OH)D was quantified by high-performance liquid chromatography-tandem mass spectrometry and categorized in 25(OH)D sufficiency ≥ 30 ng/mL, insufficiency < 30 ng/mL, and deficiency < 20 ng/mL. Therefore, the serum level of 25(OH)D was considered the best biomarker of vitamin D metabolic status and reflected contributions from all sources of vitamin D. Patients were divided into three groups: complete asthma control (ACT = 25), partial asthma control (ACT 21–24), and poor asthma control (ACT < 20).

Data were analyzed using GraphPad Prism 6 (GraphPad Software, La Jolla, CA, USA). Data description was primarily based on means and standard deviations (SD) for continuous endpoints and on frequencies for categorical parameters. Nonparametric comparisons between asthma exacerbation and asthma control were made using Wilcoxon matched-pairs signed test. The Mann–Whitney test is a method to compare two population means that come from the same population. We assessed the association between serum vitamin D levels and ACT that was evaluated by Spearman's correlation. A *P* value < 0.05 was considered statistically significant.

### 2.1. Study Population

A total of 47 adult patients with exacerbation asthma were enrolled and baseline characteristics are shown in Table 1. The mean age of patients was 63.48 ± 11.79 years. The majority of patients were aged more than 70 years (34.04%). A total of 13 of 16 (81.25%) patients with age >70 years had abnormal vitamin D levels. Of these, three patients had a vitamin D level <10 ng/mL. Subjects were predominantly female (74.47%). Also, females had more abnormal D levels (82.85%) than males (41.66%). Thirty-four subjects (72.34%) with asthma exacerbation had vitamin D levels below normal. Therefore, patients that had uncontrolled asthma (ACT < 20) and vitamin D deficiency constituted a third of the total. In the healthy control group, females comprised 65% and the mean age was 63 ± 17.29 years. The prevalence of vitamin D insufficiency in the healthy control group was 47.5% without vitamin D deficiency.

### 2.2. Serum Vitamin D Levels, Age, Sex, PEFR, and ACT Score

**Figure 1:**
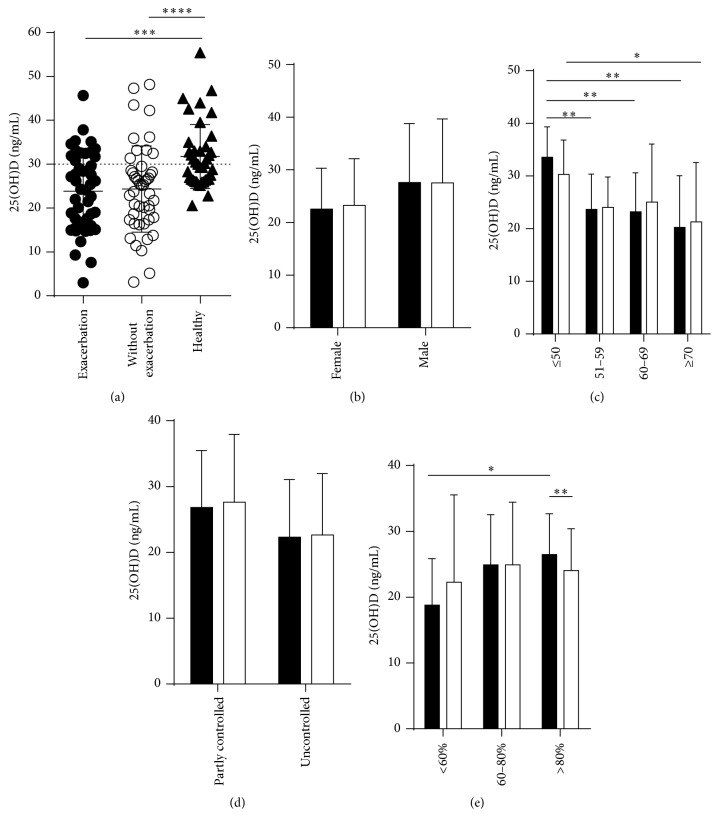
Serum vitamin D levels among severe asthma exacerbation patients. (a) The mean 25(OH)D ± SD concentrations were compared between patients with severe exacerbation, patients without asthma exacerbation for at least two weeks, and healthy controls. The mean 25(OH)D ± SD concentrations compared between during exacerbation (■, black) and without exacerbation (□, clear) of difference in sex (b), age (c), asthma control test score (d), and % predicted PEFR (e) were shown. The data were analyzed by Mann–Whitney test. ^*∗*^ *P* < 0.05, ^*∗∗*^ *P* < 0.01, ^*∗∗∗*^ *P* < 0.001, and ^*∗∗∗∗*^ *P* < 0.0001.

Mean 25(OH)D concentrations were 23.84 ± 8.89 ng/mL in the period of asthma exacerbation and increased to 24.34 ± 9.8 ng/mL in terms of no exacerbation but differed insignificantly, *P* = 0.6 (Figure 1(a)). In the healthy control group, mean 25(OH)D concentration was 31.75 ± 7.28 ng/mL. To compare between asthmatic patients and healthy controls, the mean 25(OH)D levels in the period with asthma exacerbation and without exacerbation in the asthma group were significantly lower than those in the healthy control group, *P* = 0.0002 and *P* < 0.0001, respectively (Figure 1(a)). Analyzing mean 25(OH)D concentrations between females, 22.55 ± 7.74 ng/mL, and males, 27.59 ± 11.16 ng/mL, revealed no significant difference (Figure 1(b)). However, females had a higher risk of developing vitamin D deficiency (OR = 5.83; 95% CI = 1.12–30.4, *P* = 0.03) and vitamin insufficiency than males (OR = 8.17; 95% CI = 1.30–51.4, *P* = 0.02). Age affected vitamin D level in this study. Severe asthma exacerbation mean 25(OH)D concentrations, found between ages ≤50, 51–59, 60–69, and ≥70 years, were 33.57 ± 5.77 ng/mL, 23.67 ± 6.68 ng/mL, 23.2 ± 7.4, and 20.24 ± 9.82, respectively. Age ≤50 years presented more significantly higher vitamin D levels than other age groups (Figure 1(c)). Also, in the period without exacerbation, age ≤50 had higher vitamin D levels than ≥70 years (Figure 1(c)). No significant difference was observed between partly controlled (ACT < 20) and uncontrolled asthma (ACT 21–24) in mean 25(OH)D concentration with asthma exacerbation and without asthma exacerbation (Figure 1(d)). For asthmatic patients with % predicted PEFR > 80%, mean 25(OH)D concentration 26.48 ± 6.17 ng/mL was significantly higher than in asthmatic patients with % predicted PEFR <60% (18.81 ± 7.04 ng/mL, *P* = 0.02) (Figure 1(e)). In patients with % predicted PEFR > 80%, mean 25(OH)D concentrations between with asthma exacerbation 26.48 ± 6.17 and without asthma exacerbation 24.34 ± 6.36 ng/mL significantly differed, *P* = 0.009 (Figure 1(e)).

**Figure 2:**
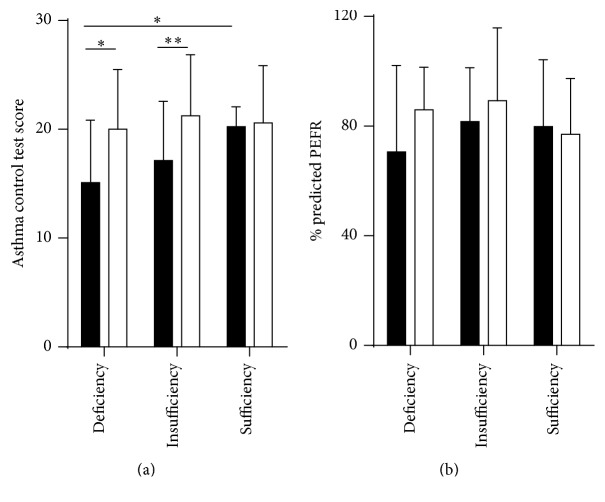
(a) The bar graph comparison between patients during asthma exacerbation (■, black bar) and without exacerbation (□, clear bar); we found difference vitamin D status affects asthma control test score. (b) Vitamin D status and % predicted PEFR. The data were analyzed by Wilcoxon matched-pairs signed test and Mann–Whitney test. ^*∗*^ *P* < 0.05, ^*∗∗*^ *P* < 0.01.

We also observed vitamin D status with controlled asthma and lung function. Representative vitamin D status and asthma control test score are shown in Figure 2(a). Interestingly, vitamin D status affected the ability to control asthma. Patients with sufficient vitamin D levels had significantly higher ACT scores than patients with vitamin D deficiency during asthma exacerbation (20.23 ± 1.83 versus 15.11 ± 5.73, *P* = 0.04) (Figure 2(a)). Moreover, during the period without asthma exacerbation, patients with vitamin D insufficiency and deficiency significantly increased asthma control test score to compare in the period with exacerbation asthma (Figure 2(a)). Vitamin D status presented no difference in % predicted PEFR (Figure 2(b)).

**Figure 3:**
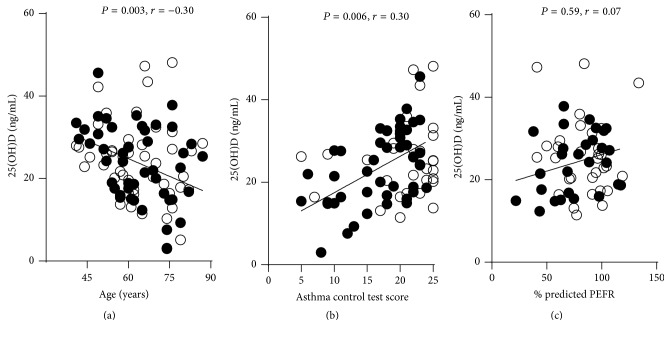
The correlation graph between age, asthma control test score, and % predicted PEFR with serum 25(OH)D levels. (a) The graph shows a negative linear correlation between age and serum 25(OH)D levels. (b) Positive linear correlation between asthma control test score and serum 25(OH)D levels. (c) Correlation between % predicted PEFR and serum 25(OH)D levels. Spearman's test was used to determine correlation. Serum vitamin D levels during exacerbation (●, black) and without exacerbation (○, clear) asthma.

We next tested whether serum vitamin D levels were associated with age, controlled asthma, and % predicted PEFR. Increasing age inversely correlated with serum vitamin D level (*P* = 0.003, *r* = −0.30) (Figure 3(a)). Then serum 25(OH)D levels significantly positively correlated with controlled asthma (*P* = 0.006; *r* = 0.30) (Figure 3(b)). However, an association between serum vitamin D levels and lung function was not observed (Figure 3(c)).

### 2.3. Supplement Vitamin D in Asthmatic Patients with Low Vitamin D Levels

**Figure 4:**
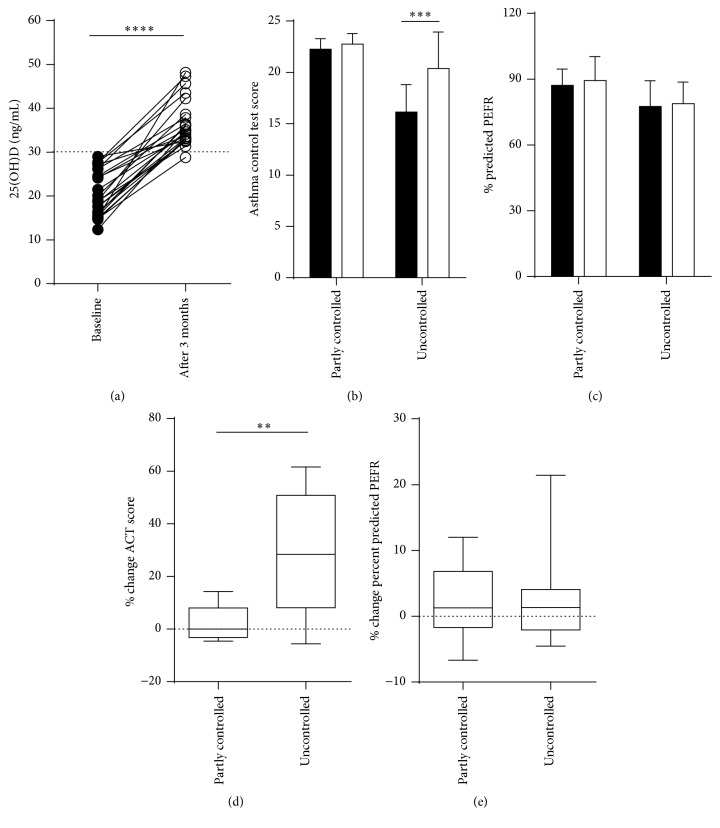
Patients with low vitamin D levels receiving vitamin D supplement for 3 months. (a) Successful vitamin D supplement, 25(OH)D concentration increased significantly after 3 months. (b) ACT scores were of significant differences among uncontrolled asthma group to compare between at baseline (■, black) and after 3 months (□, clear) of vitamin D2 supplement. (c) % predicted PEFR with partly controlled (■, black) and uncontrolled asthma (□, clear). % change ACT score (d) and % change percent predicted PEFR (e) compared between partly controlled and uncontrolled asthma group. The data were analyzed by Wilcoxon matched-pairs signed test and Mann–Whitney test. ^*∗∗*^ *P* < 0.01, ^*∗∗∗*^ *P* < 0.001, and ^*∗∗∗∗*^ *P* < 0.0001.

Twenty-four of 34 subjects (70.58%) who had serum 25(OH)D level less than 30 ng/mL participated in receiving vitamin D2 supplement (12 subjects denied treatment with vitamin D supplement). We prescribed a vitamin D2 supplement, ergocalciferol (vitamin D2), 20,000 IU every alternate day for 3 months to 24 asthmatic patients (7, partly controlled, and 17, uncontrolled asthma). After 3 months of vitamin D administration, mean 25(OH)D concentration was significantly higher than baseline among asthmatic patients (20.41 ± 5.13 versus 36.41 ± 5.22 ng/mL, *P* < 0.0001) (Figure 4(a)). To compare with baseline, vitamin D supplement improved asthma control in uncontrolled asthmatic patients. In the uncontrolled asthma group, mean ACT score at baseline, 16.12 ± 1.03, significantly increased after receiving vitamin D supplement for 3 months (20.37 ± 3.55, *P* = 0.0004) (Figure 4(b)). The lung function in the partly controlled and uncontrolled asthma group showed slightly increased % predicted PEFR after 3 months of vitamin D supplement but without significant difference (Figure 4(c)). Next, we observed % change of ACT score and percent predicted PEFR between partly controlled and uncontrolled asthma group. Uncontrolled asthma group had significantly improved ACT score more than partly controlled asthma group (Figure 4(d)). No significant difference with % change of percent predicted PEFR between both groups was observed (Figure 4(e)). Furthermore, no side effect of vitamin D supplement was reported from this study.

## 3. Discussion

This cross-sectional study examined vitamin D levels in adult asthmatic patients with differing periods of severe asthma exacerbation and without exacerbation. The study could not conclude that decreased vitamin D level was the leading cause of severe asthma exacerbation among asthmatic Thai adults. However, many studies have suggested vitamin D deficiency was linked to increased risk of asthma exacerbation. Vitamin D insufficiency among North American and Puerto Rican children was associated with severe asthma exacerbations [25, 26]. Moreover, adults presenting vitamin D deficiency were reported in to be linked to the risk of severe asthma exacerbations [27]. Furthermore, a large cohort from Israel showed asthmatic patients with vitamin D deficiency had a 25% higher chance of having an exacerbation than patients within normal range [28]. However, many studies examined serum vitamin D level only one time and did not compare with the period without exacerbation in the same patient.

We showed vitamin D insufficiency and deficiency were more highly prevalent among asthmatic patients than among healthy people. The prevalence of low vitamin D status was 45% among healthy Thai people [29]. Similarly, the study in asthmatic Thai children showed the prevalence of decreased vitamin D status was 64% [30]. Thailand, a Southeast Asian country, is located close to the equator and has a tropical rainforest climate with high sun exposure, one source of vitamin D among humans. One possible explanation is that vitamin D metabolism is influenced by various factors including skin pigmentation, sunscreen use, clothing, drugs, smoking, age, obesity, and several chronic illnesses. Dietary intake and supplements are a secondary source of vitamin D. Few natural vitamin D rich food sources are available in Thailand and foods are not fortified with vitamin D [31]. Avoiding sun exposure and low intake of vitamin D may play significant roles in vitamin D insufficiency among people.

An inverse relationship was found between concentration of 25(OH)D and age. The frequency of vitamin D insufficiency was more common in females and elderly patients. Being old and female might influence vitamin D levels in asthmatic patients. The study by Chailurkit et al. among 2,641 Thai adult subjects found the prevalence of vitamin D insufficiency was dependent on geographic region and associated with females, younger age, and living in an urban area [32]. A positive relationship was observed between concentrations of 25(OH)D and asthma control test score. The findings of the present study confirmed report that patients with various degrees of vitamin D status were associated with asthma severity, monitored among children and adults [27, 33]. Lower vitamin D levels were associated with worse asthma control.

Asthma has been treated by vitamin D supplement in at least three adult random controlled trials (RCT). Arshi et al. conducted an RCT enrolling 130 patients with moderate persistent asthma and found vitamin D supplementation to be associated with asthma controllers that could significantly improve FEV1 in mild to moderate persistent asthma after 24 weeks [34]. In addition, Yadav and Mittal reported monthly doses of 60,000 IU vitamin D3 significantly reduced the number of exacerbations as compared with placebo and PEFR significantly increased in the treatment group [35]. Moreover, the study by De Groot et al. conducted an RCT involving 44 patients with nonatopic asthma and found vitamin D supplementation reduced eosinophilic airway inflammation among patients with nonatopic asthma with severe eosinophilic airway inflammation [36]. However, Castro et al. studied RCT involving 408 adult asthmatic patients and concluded vitamin D3 did not reduce the rate of exacerbation among adults with persistent asthma and vitamin D insufficiency [37]. In addition, Martineau et al. conducted an RCT comprising 250 adults with asthma in London and reported bolus-dose vitamin D3 supplementation did not influence time to exacerbation in a population of adults with asthma [38]. The more recent RCT results suggest likely interactions of the vitamin D acute respiratory tract infection association by baseline vitamin D status (i.e., vitamin D deficient individuals receive more benefit than those with normal concentrations) and possibly greater advantage for subgroups that are at higher risk of acute respiratory tract infection, such as young children, older adults, or individuals with immune defects [39]. Providing vitamin D supplements to improve asthma symptoms and treatment is controversial.

The limitation of the study is no randomized control trial and no control group in vitamin D supplement study that it is difficult to conclude the benefit of vitamin D supplement in uncontrolled asthmatic patients. It might be a placebo effect. In addition, the subjects in this study are of low number because asthma exacerbation with upper respiratory tract infection is common in Thailand; this study excluded the cause of asthma exacerbation with upper respiratory tract infection. We used vitamin D2 supplementation because the cost of the treatment is less than vitamin D3 supplementation. However, vitamin D2 supplementation is effective in increasing serum 25(OH)D level and not toxic when provided in large amounts. Further study will be planned to do randomized control trial and multicenter study to prove the benefit of vitamin D supplement in asthmatic patients.

## 4. Conclusion

Hypovitaminosis D was common in patients with asthma exacerbation, but the low vitamin D level did not cause asthma exacerbation. Vitamin D insufficiency and deficiency were usually found in females and the elderly. Lower vitamin D level increased the difficulty in controlling asthma. Vitamin D supplements provided to uncontrolled asthmatic patients help to control asthma symptoms. The study showed the benefits of examining serum 25(OH)D and providing vitamin D supplements to uncontrolled asthmatic patients.

## Figures and Tables

**Table 1:** Baseline characteristics of patients with asthma exacerbation and healthy subjects.

Characteristics	Vitamin D level (*n* = 47)			Healthy (*n* = 40)	
	Deficiency	Insufficiency	Sufficiency	Insufficiency	Sufficiency
Age, *n* (%)					
<50 years	0 (0)	2 (4.26)	5 (10.64)	2 (5.00)	5 (12.5)
50–59 years	4 (8.51)	4 (8.51)	2 (4.26)	4 (10.00)	8 (20.00)
60–69 years	6 (12.77)	5 (10.64)	3 (6.38)	6 (15.00)	6 (15.00)
>70 years	8 (17.02)	5 (10.64)	3 (6.38)	7 (17.50)	2 (5.00)
Gender, *n* (%)					
Female	15 (31.91)	14 (29.79)	6 (12.77)	12 (30.00)	14 (35.00)
Male	3 (6.38)	2 (4.26)	7 (14.89)	7 (17.50)	7 (17.50)
Asthma control, *n* (%)					
Partly controlled	2 (4.26)	5 (10.64)	7 (14.89)	0 (0)	0 (0)
Uncontrolled	16 (34.04)	11 (23.4)	6 (12.77)	0 (0)	0 (0)

## Abbreviations

25(OH)D: 25-Hydroxyvitamin D
ACT: Asthma control test
PEFR: Peak expiratory flow rate.
